# Joint association of the frailty index and phenotypic age with all-cause and cause-specific mortality: A prospective cohort study

**DOI:** 10.1515/jtim-2026-0046

**Published:** 2026-06-13

**Authors:** Zilun Shao, Yuxuan Zhao, Haiming Yang, Meng Xiao, Mingyu Song, Chunxiao Liao, Yuanjie Pang, Wenjing Gao, Tao Huang, Canqing Yu, Shengxu Li, Lu Qi, Liming Li, Jun Lv, Dianjianyi Sun

**Affiliations:** Department of Epidemiology and Biostatistics, School of Public Health, Peking University Health Science Center, Beijing, China; Peking University Center for Public Health and Epidemic Preparedness & Response, Beijing, China; Key Laboratory of Epidemiology of Major Diseases (Peking University), Ministry of Education, Beijing, China; Children's Minnesota Research Institute, Children's Minnesota Hospital, Minneapolis, MN, USA; Department of Epidemiology, School of Public Health and Tropical Medicine, Tulane University, New Orleans, LA, USA; Department of Nutrition, Harvard T.H. Chan School of Public Health, Boston, MA, USA; State Key Laboratory of Vascular Homeostasis and Remodeling, Peking University, Beijing, China

**Keywords:** frailty index, phenotypic age, mortality risk

## Abstract

**Background and Objectives:**

Little is known about whether frailty index (FI) and phenotypic age (PhenoAge), two independent indicators of biological age, may jointly or interactively predict death risk.

**Methods:**

This prospective study was conducted using National Health and Nutrition Examination Survey (NHANES) data of four survey cycles (2003 to 2010) and mortality records until December 31, 2019. FI was calculated for each participant based on his/her responses to 34 questions, and PhenoAge was derived from algorithms using data of nine biomarkers and chronological age. We used Cox proportional hazards (PH) models and competing risk models to estimate the associations of FI, PhenoAge, and their interactions with all-cause mortality and cause-specific mortality. The C-statistic, continuous net reclassification index (NRI), and integrated discrimination improvement (IDI) were used to evaluate model performance.

**Results:**

After an updated median follow-up of 11.8 years among 20, 089 adults, a total of 3778 deaths were documented. Each 1 standard deviation (SD) increment in FI and PhenoAgeAccel was associated with 47% (42%-51%) and 41% (38%-45%) higher risk of all-cause mortality, respectively. However, their multiplicative interaction on death risk was not found (*P* = 0.054). Compared with the reference group (robust-decelerated aging), the risk of all-cause mortality was the highest in the frail-accelerated aging group with a hazard ratio (HR) (95% confidence interval [CI]) of 5.32 (4.50-6.29). Notably, adding both FI and PhenoAgeAccel to the traditional model significantly enhanced predictive accuracy (C-index 0.882 *vs*. 0.866, NRI 46.18%, and IDI 4.10%).

**Conclusions:**

Individual death risk could be better predicted by a joint inclusion of both FI and PhenoAge, indicating that targeting their components may reduce death risk and prolong health span.

## Introduction

Aging is a universal risk factor for death and chronic diseases, including cardiovascular, cancerous, metabolic, and neurological diseases.^[1,2]^ Individuals may share the same chronological age, but the age-related functional decline in organs and the body can vary significantly.^[3]^ Hence, biological age is now acknowledged as a more efficient marker, enabling early identification of individuals at increased risk of death and age-related diseases.

Frailty index (FI) is a frequently utilized clinical indicator for assessing functional aging,^[4]^ which is measured as the proportion of accumulated deficits and defines frailty by predefined cut-points.^[5]^ It has been demonstrated as an effective predictor of adverse outcomes, including mortality, in various populations. However, while FI has been extensively studied, most research focuses on using it as a singular measure, with limited attention given to how it might interact with other aging-related markers.

Phenotypic age, which reflects an individual’s biological age based on multiple biomarkers and clinical parameters, has also been identified as a reliable predictor of mortality risk.^[6]^ Unlike chronological age, phenotypic age takes into account the degree of functional decline and accumulated health deficits, offering a more dynamic and individualized approach to risk prediction. FI and phenotypic age are representative clinical predictors that have been confirmed for mortality in the population.^[7, 8, 9, 10]^ However, most studies have primarily focused on evaluating these predictors individually, and little is known on their joint association with mortality risk or their potential interactions. The limitations of this approach lie in the fact that it overlooks the potential interaction between these two measures of aging. For instance, while each of these indices is independently associated with mortality, little research has explored whether their combined effect offers greater predictive value. Additionally, there is a gap in understanding whether the two indices capture different or overlapping aspects of the aging process, which could inform more accurate risk assessments for individuals.

In this study, we prospectively examined the associations of FI and phenotypic age with mortality (all-cause and cause-specific), and further evaluated whether the FI and phenotypic age, individually or combined, had added value to the mortality risk prediction.

## Methods

### Data sources and study population

Data for this prospective study was extracted from the National Health and Nutrition Examination Survey (NHANES). NHANES consists of a series of cross-sectional surveys that assess the health and nutritional status of adults and children in the United States. A detailed description of the study design, protocol, and data collection methods was provided in https://www.cdc.gov/nchs/nhanes/index.htm.

In this study, four NHANES survey cycles (2003-2004, 2005-2006, 2007-2008, and 2009-2010) were included (*n* = 41,156). We excluded participants under the age of 20 (*n* = 18,983), those with missing biomarker data for phenotypic age or missing values for items used to construct the FI (*n* = 2023), and with missing data on follow-up outcomes (*n* = 61). A total of 20,089 adults were included in the final analytic sample.

The NHANES survey protocol was approved by the Institutional Review Board of the Centers for Disease Control and Prevention, and all participants provided written informed consent.

### Phenotypic age and phenotypic age acceleration

Phenotypic age (PhenoAge) represents the biological age within the general population that aligns with an individual’s risk of mortality.^[6,7]^ According to the procedure proposed by Levine, we calculated PhenoAge using chronological age and nine routine biomarkers (Supplementary Table S1).^[6]^

Phenotypic age acceleration (PhenoAgeAccel) is calculated as a residual value derived from regressing PhenoAge values on chronological age using linear regression, providing a measure of phenotypic aging independently of chronological age.^[6]^ Based on PhenoAgeAccel, we further categorized the individuals into three levels: decelerated aging (PhenoAgeAccel ≤ -5), normal aging (-5 < PhenoAgeAccel < 5), and accelerated aging (PhenoAgeAccel ≥ 5).

### FI

FI is the proportion of deficits present in physical functions at the time of health appraisal.^[5]^ In the present study, we constructed FI using 34 baseline variables involving disease histories, physical functions, and symptoms (Supplementary Table S2). This 34-item FI was adapted from a previously validated FI in NHANES^[11]^ and ranged from 0 to 1, with a higher score indicating a frailer condition. In light of previous studies,^[12]^ we divided FI into three levels of frailty: robust (FI ≤ 0.10), prefrail (0.10 < FI < 0.25), and frail (FI ≥ 0.25).

### Procedures

Sociodemographic characteristics (age, sex, race, marital status, family income, and level of education) and lifestyle factors (smoking status and alcohol consumption) were collected with questionnaires at survey interviews. The assessment of physical activity involved multiplying the metabolic equivalent tasks (METs) values of activities by the standard daily duration of these activities in hours. Dietary factors from the dietary datasets were used to calculate the healthy eating score (HEI).^[13]^ Information on comorbidities was acquired from questionnaire data that contained self-reported and physician-diagnosed diseases. Participants who reported physician-diagnosed congestive heart failure, coronary heart disease, angina/ angina pectoris, heart attack, or stroke were defined as having cardiovascular disease (CVD). Those who reported having either type of cancer were defined as having cancer. For each participant, the blood sample was collected in the Mobile Examination Center. On-site pregnancy testing excluded pregnant women from other examination components such as Dual-emissionX-ray Absorptiometry. All other specimen testing was performed by Federal, private, and university-based laboratories under contract to the National Center for Health Statistics (NCHS).

### Outcomes

All-cause and cause-specific mortality information were determined by the NHANES-linked National Death Index datasets through December 31, 2019. Causes of death were classified using the 10th revision of International Classification of Diseases (ICD-10) codes. The primary outcomes of this study included all-cause mortality, CVD mortality (I00-I09, I11, I13, I20-I51, and I60-I69), and cancer-related mortality (C00-C97).

### Statistical analysis

Baseline characteristics of the study population were presented by three categories of PhenoAgeAccel. We compared differences among groups using analysis of variance for continuous variables and the *chi-square* test for categorical variables. We calculated the follow-up person-years from the date participants completed the baseline survey to the date of death, loss to follow-up, or December 31, 2019, whichever came first.

We used multivariable Cox proportional hazards (PH) models to estimate hazard ratios (HRs) and 95% confidence intervals (CIs) for the associations of the FI and PhenoAgeAccel with all-cause mortality. Fine and Gray’s competing risk models^[14]^ were used to estimate HRs and 95% CIs for the associations of the FI and PhenoAgeAccel with cause-specific mortality. To assess the proportional hazards assumption for the Cox regression model, we conducted the Schoenfeld residuals test for each covariate. No significant violations of the PH assumption were found. All models were adjusted for sex (male and female), age (years), ethnicity (non-Hispanic white, non-Hispanic black, Mexican American, other Hispanic, and others), marital status (married, widowed, divorced/ separated, and never married), education (less than high school, high school, some college/ associate education and college graduate or more), family income (<$20,000, $20,000 – <$45,000, $45,000 – <$75,000, and ≥$75,000), smoking status (never, former, current), excessive alcohol use (no and yes), physical activity (none, failing to achieve recommended levels, and achieving recommended levels), HEI scores, and body mass index (BMI; kg/m^2^), with additional adjustment for baseline comorbidities of CVD or cancer in the corresponding cause-specific analyses. The Wald test for linear trend was used to assess a linear trend across the multilevel variables. To investigate the joint association between the FI and PhenoAgeAccel on the mortality risk, we classified participants into nine groups according to their joint categories, with robust-decelerated aging as the reference group. To assess the absolute risks for different joint groups, we computed the 10-year cumulative incidence and calculated the absolute risk differences between the different joint groups. Additive interactions were estimated using relative excess risk due to interaction (RERIs) and attributable proportion (APs) due to interaction with their 95% CIs (estimated by variance recovery method) by fitting cox hazard models with an interaction term for FI and PhenoAgeAccel on all-cause and cause-specific deaths. Multiplicative interactions were tested by comparing the multivariate-adjusted models with and without cross-product interaction terms using likelihood-ratio tests. We performed a sensitivity analysis by excluding those who had died within two years of the baseline survey to minimize any potential reverse causality.

We applied C-index, net reclassification index (NRI), and integrated discrimination improvement (IDI) to evaluate the added value of FI and PhenoAgeAccel in risk prediction of mortality. The C-index offers a comprehensive statistical evaluation of survival model for continuous event times.^[15]^ In contrast to C-index values, NRI and IDI illustrate the percentage of a population that is accurately reclassified, providing clinically meaningful results rather than only statistically significant differences.^[16]^

All analyses were performed using R version 4.1.2, and statistical significance was set at a two-tailed *P* < 0.05.

## Results

### Population characteristics

A total of 20,089 participants were included in the analysis; 48.4% were males, and the average age was (49.7 ± 18.5) years. There were 4472 (22.3%) participants with decelerated aging, 12,027 (59.9%) with normal aging, and 3590 (17.8%) with accelerated aging (Table 1). Compared with the other two groups, participants in the accelerated aging group were more likely to be non-Hispanic black, have lower levels of education and household income, be ever smokers, engage in lower levels of physical activity, and have lower HEI scores and higher BMI.

**Table 1:** Baseline characteristics of the study participants by PhenoAgeAccel status (*_n_* = 20,089)

Characteristics		Decelerated aging (*n* = 4472)	Normal aging (*n* = 12,027)	Accelerated aging (*n* = 3590)
Sex, *n* (%)	Male	1709 (38.2)	6294 (52.3)	1714 (47.7)
	Female	2763 (61.8)	5733 (47.7)	1876 (52.3)
Age, *n* (%)	20-39 years	1177 (26.3)	4666 (38.8)	1105 (30.8)
	40-65 years	1953 (43.7)	4698 (39.1)	1394 (38.8)
	≥ 65 years	1342 (30.0)	2663 (22.1)	1091 (30.4)
Race/ethnicity, *n* (%)	Non-Hispanic white	2408 (53.8)	5992 (49.8)	1581 (44.0)
	Non-Hispanic black	620 (13.9)	2429 (20.2)	969 (27.0)
	Mexican American	803 (18.0)	2307 (19.2)	663 (18.5)
	Other Hispanic	398 (8.9)	817 (6.8)	237 (6.6)
	Others	243 (5.4)	482 (4.0)	140 (3.9)
Marital status, *n* (%)	Married	2637 (62.6)	6334 (57.2)	1723 (52.1)
	Widowed	481 (11.4)	937 (8.5)	426 (12.9)
	Divorced/separated	515 (12.2)	1599 (14.4)	610 (18.5)
	Never married	578 (13.7)	2203 (19.9)	545 (16.5)
Education, *n* (%)	Less than high school	1129 (25.3)	3405 (28.3)	1267 (35.4)
	High school	961 (21.5)	2984 (24.8)	905 (25.3)
	Some college/associate education	1203 (26.9)	3351 (27.9)	958 (26.7)
	College graduate or more	1174 (26.3)	2274 (18.9)	454 (12.7)
Family income (USD/year), *n* (%)	<20,000	780 (18.9)	2547 (22.7)	1049 (31.4)
	20,000 – <45,000	1291 (31.3)	3662 (32.7)	1195 (35.7)
	45,000 – <75,000	878 (21.3)	2384 (21.3)	619 (18.5)
	≥75,000	1173 (28.5)	2603 (23.2)	481 (14.4)
Smoking status, *n* (%)	Never	2735 (61.2)	6172 (51.4)	1620 (45.1)
	Former	1222 (27.3)	2904 (24.2)	1005 (28.0)
	Current	515 (11.5)	2943 (24.5)	964 (26.9)
Excessive alcohol use^*^, *n* (%)	No	3573 (88.6)	9331 (87.4)	2883 (91.7)
	Yes	458 (11.4)	1343 (12.6)	260 (8.3)
Physical activity^†^, *n* (%)	None	1946 (43.5)	5704 (47.5)	2175 (60.6)
	Fail to achieve recommended levels	909 (20.3)	2283 (19.0)	624 (17.4)
	Achieve recommended levels	1614 (36.1)	4031 (33.5)	788 (22.0)
HEI, (mean ± SD, scores)		52.93 ± 14.91	48.14 ± 14.20	47.25 ± 14.05
BMI, (mean ± SD, kg/m^2^)		26.22 ± 4.70	28.96 ± 6.25	32.10 ± 8.41
Comorbidities, *n* (%)	CVD	1488 (33.4)	4215 (35.2)	1905 (53.3)
	Cancer	417 (9.3)	1069 (8.9)	416 (11.6)
Frailty status^‡^, *n* (%)	Robust	2875 (64.3)	7824 (65.1)	1532 (42.7)
	Prefrail	1277 (28.6)	2976 (24.7)	1166 (32.5)
	Frail	320 (7.2)	1227 (10.2)	892 (24.8)

^*^Excessive alcohol use was defined as having 14 g/d of pure alcohol for women (1 standard alcoholic drink/d) and 28 g/d for men (2 standard alcoholic drinks/d). ^†^Physical activity levels were classified according to physical activity guidelines (150 min/wk of moderate-intensity physical activity, 75 min/wk of vigorous-intensity physical activity, or an equivalent combination). ^‡^Frailty status was categorized into three levels: robust (FI ≤ 0.10), prefrail (0.1 < FI < 0.25), and frail (FI ≥ 0.25). HEI: healthy eating index; BMI: body mass index; CVD: cardiovascular disease; SD: standard deviation.

**Figure 1:**
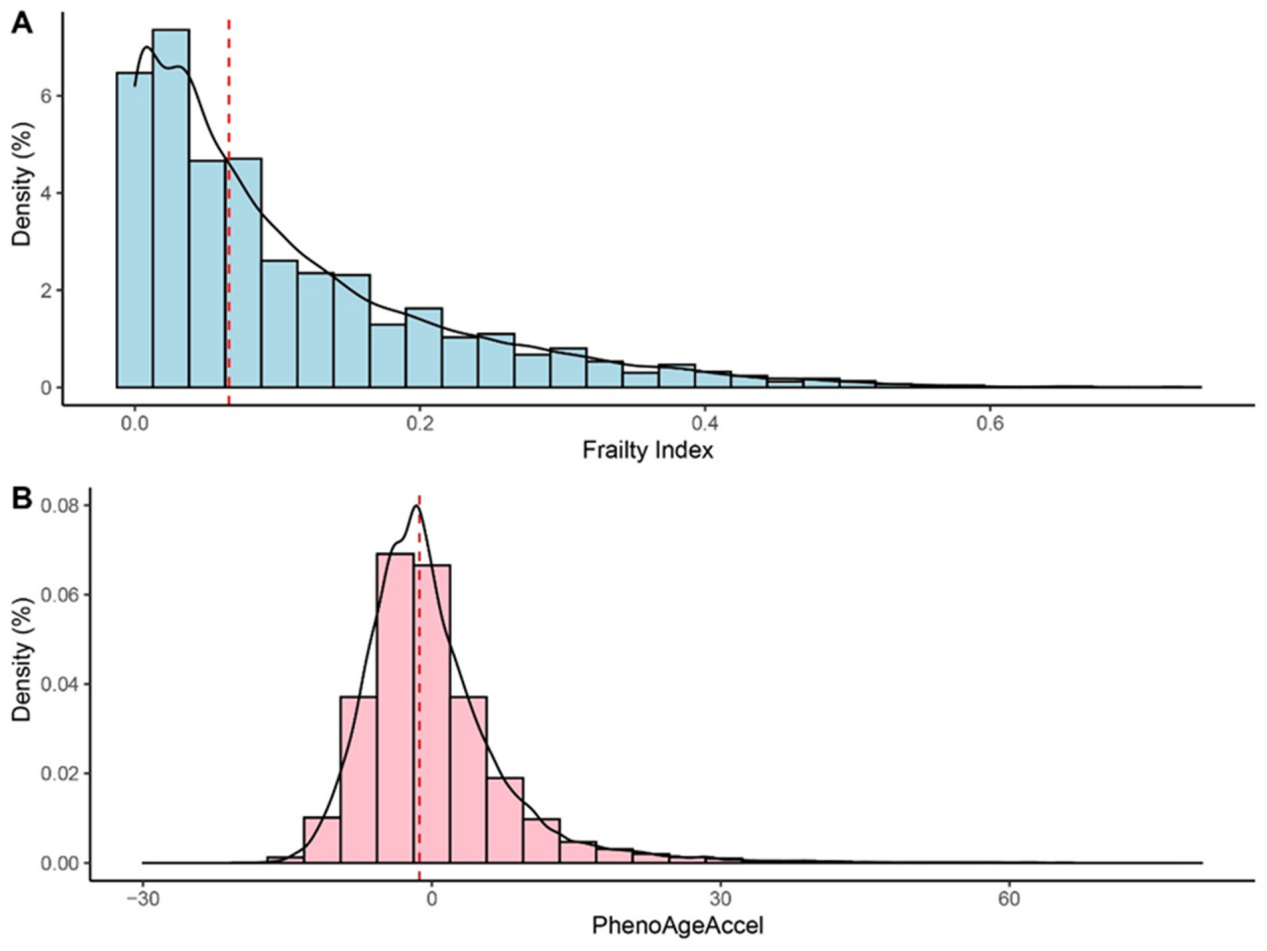
Distribution of frailty index and PhenoAgeAccel at baseline.

The distribution of the FI exhibited positive skewness, and it had a median value of 0.066 (interquartile range: 0.125) and a mean of 0.107 (Figure 1). PhenoAgeAccel distribution was also positively skewed, with the majority of outliers in the positive (older) direction. The median PhenoAgeAccel across all participants was -1.290 (interquartile range: 7.607), with a mean of 0.083.

### Associations of FI and PhenoAge with all-cause and cause-specific mortality

During a median follow-up of 11.8 years, 3778 deaths (2124 males and 1654 females) were recorded, including 1195 deaths from CVD and 859 deaths from cancer. According to the Kaplan-Meier curve of survival probability by frailty or PhenoAgeAccel status, those with the worst health status at baseline (frail based on FI or accelerated aging based on PhenoAgeAccel) had the lowest survival probability during follow-up.

**Figure 2:**
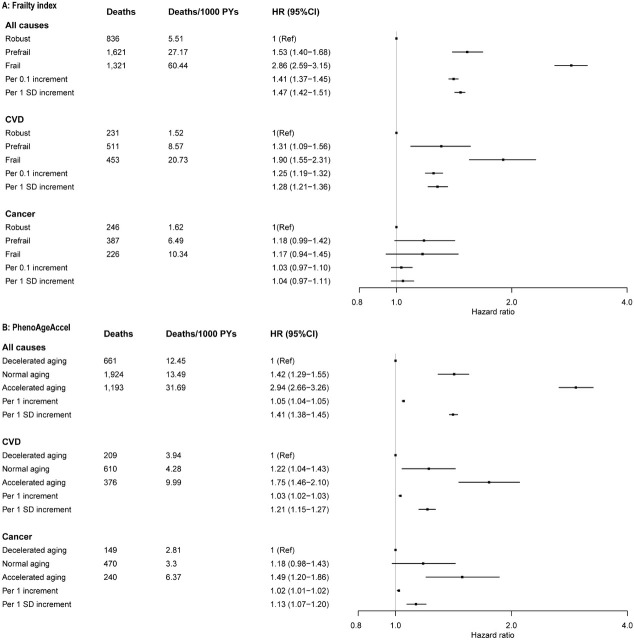
The association of frailty status and PhenoAge status with all-cause and cause-specific mortality. (A) Association of frailty status with all-cause and cause-specific mortality. (B) Association of PhenoAge status with all-cause and cause-specific mortality. Multivariable models were adjusted for age, sex, ethnicity, marital status, education level, family income, smoking status, alcohol use, physical activity, healthy eating index score, BMI, and baseline prevalence of CVD and cancer (only in corresponding cause-specific analyses). All *P* for trend < 0.001. PYs: person-years; HR: hazard ratio; CI: confidence interval; Ref: reference; SD: standard deviation; BMI: body mass index; CVD: cardiovascular disease.

After adjusting for known and potential confounders, FI and PhenoAgeAccel were both independently associated with an increased risk of all-cause mortality (Figure 2). For FI, compared with the robust group, the HRs (95% CIs) were 1.53 (1.40-1.68) and 2.86 (2.59-3.15) for the prefrail and frail groups, respectively, with the HR (95% CI) per 0.1 increment in FI of 1.41 (1.37-1.45) and 1 standard deviation (SD) increment in FI of 1.47 (1.42-1.51). For PhenoAgeAccel, compared with the decelerated aging group, the HRs (95% CIs) were 1.42 (1.29-1.55) and 2.94 (2.66-3.26) for the normal aging and accelerated aging groups, respectively, with the HR (95% CI) 1 increment in PhenoAgeAccel of 1.05 (1.04-1.05) and 1 SD increment in PhenoAgeAccel of 1.41 (1.38-1.45). The FI and PhenoAgeAccel were also associated with increased risks of death from CVD. Additionally, PhenoAgeAccel was associated with increased risk of cancer mortality, whereas no statistically significant association was observed between FI and cancer mortality. For each 1 SD increment in FI, the corresponding HR (95%CI) was 1.28 (1.21-1.36) for CVD death and 1.04 (0.97-1.11) for cancer death. For each 1 SD increment in PhenoAgeAccel, the corresponding HRs (95% CIs) for risk of death from CVD and cancer were 1.21 (1.15-1.27) and 1.13 (1.07-1.20), respectively.

In sensitivity analyses, after excluding participants who died within two years of the baseline survey, the association of FI and PhenoAgeAccel with all-cause and cause-specific mortality showed only slight changes (Supplementary Table S3).

### Joint association of FI and PhenoAge on the risk of mortality

Our study revealed a weak correlation between PhenoAge and FI (*r* = 0.24, *P* < 0.001). There was no statistically multiplicative significant interaction between the FI and PhenoAgeAccel status on all-cause mortality (*P* for interaction = 0.054; Supplementary Table S4). Similarly, no significant multiplicative interactions were observed for CVD or cancer (*P* for interaction = 0.743 and 0.157, respectively). In contrast, a significant additive interaction between FI and PhenoAgeAccel was identified for all-cause mortality (RERI = 0.148, 95% CI: 0.078-0.219; AP = 0.265, 95% CI: 0.109-0.422, *P* < 0.01). No significant additive interaction was found for CVD or cancer.

**Figure 3:**
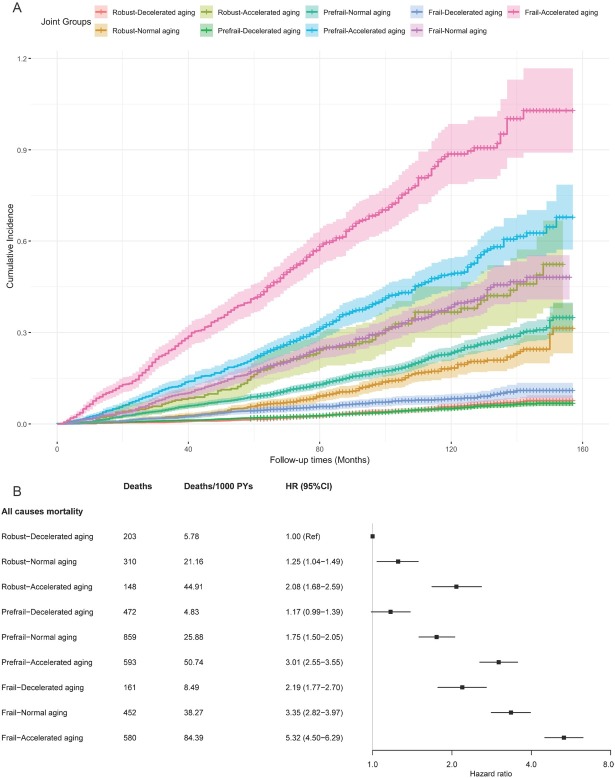
Cumulative incidence and hazard ratios for all-cause mortality by joint classification of frailty status and PhenoAgeAccel. (A) Adjusted cumulative incidence curves for all-cause mortality stratified by frailty status and PhenoAgeAccel joint groups. (B) The joint association of frailty status and PhenoAgeAccel joint groups with all-cause mortality.Multivariate models were adjusted for age, sex, ethnicity, marital status, education level, family income, smoking status, alcohol use, physical activity, healthy eating index score, and BMI. PYs: Person-years; HR: hazard ratio; CI: confidence interval; Ref: reference.

The cumulative incidence curves are shown in Figure 3A, clearly illustrating the increased mortality risk in individuals with accelerated aging phenotypes, particularly in the frail-accelerated aging group. In Figure 3B, in comparison to the reference group (robust-decelerated aging category), the frail-accelerated aging group showed the highest risk (5.32, 4.50-6.29). Additionally, the 10-year absolute risk for this group was 53.26% (95% CI: 49.05-57.47), the highest among all the groups (Supplementary Table S5). The association results between the joint categories and the risk of cause-specific mortality were shown in the supplementary materials (Supplementary Figure S1).

In sensitivity analyses, after excluding participants who died within two years of the baseline survey, the joint association of FI and PhenoAgeAccel with the risk of mortality was not materially altered (Supplementary Table S6).

### Added value of FI and PhenoAge in mortality risk prediction improvement

The addition of FI and PhenoAgeAccel, individually or combined, to a conventional mortality risk prediction model increased the C-index, continuous NRI, and IDI (Table 2). The C-index for the model including both FI and PhenoAgeAccel was 0.882 (0.877-0.887). The model that included both FI and PhenoAge also performed better than the models that included either FI or PhenoAgeAccel alone (Supplementary Table S7).

**Table 2:** Added value of FI and PhenoAgeAccel in risk prediction improvement

Model	C-index (95% CI)	*P*	NRI, continuous (95% CI), %	*P*	IDI (95% CI), %	*P*
Conventional model	0.866 (0.861-0.871)	-	-	-	-	-
Conventional model + FI	0.875 (0.870-0.880)	< 0.001	41.19 (32.61-48.38)	< 0.001	2.30 (1.80-2.80)	< 0.001
Conventional model + PhenoAgeAccel	0.877 (0.872-0.882)	< 0.001	33.05 (26.58-41.75)	< 0.001	2.60 (2.20-3.00)	< 0.001
Conventional model + FI +PhenoAgeAccel	0.882 (0.877-0.887)	< 0.001	46.18 (36.59-54.68)	< 0.001	4.10 (3.50-4.70)	< 0.001

Conventional model included age, sex, ethnicity, marital status, education level, family income, smoking status, alcohol use, physical activity, healthy eating index score, and BMI. CI: confidence interval; NRI: net reclassification improvement; IDI: integrated discrimination index.

## Discussion

In this United States (US) adult prospective cohort, we found that FI and PhenoAgeAccel were independently associated with increased risks of all-cause and CVD mortality, and PhenoAgeAccel, but not FI, was associated with an increased risk of cancer mortality. Importantly, combining FI and PhenoAgeAccel improved the predictive performance of mortality compared with the conventional prediction model or the model with further addition of either alone. FI and PhenoAge showed different aspects of health, with FI reflecting the level of frailty and PhenoAge measuring the rate of aging. Although the underlying biological mechanisms of frailty and aging differ, both conditions involve a decline in the physiological reserve of the body.^[17]^ Previous studies have consistently demonstrated that both FI and PhenoAge are strong predictors of all-cause mortality. In a study based on data from the Epidemiologische Studie zu Chancen der Verhütung, Früherkennung und Therapie chronischer Erkrankungen in der älteren Bevölkerung (ESTHER) in Germany, the HR (95% CI) for all-cause mortality was 1.31 (1.20-1.43) and 1.32 (1.19-1.46) per one SD increase of FI and PhenoAgeAccel, respectively.^[18]^ Furthermore, a meta-analysis of 19 prospective studies suggested that frailty measured by FI is a significant predictor of mortality, with an HR (95% CI) of 1.28 (1.29-1.31) for all-cause mortality.^[9]^ Our findings are consistent with these previous studies. However, research on cause-specific mortality is limited. Available studies consistently showed that FI was associated with an increased risk of mortality from CVD. ^[4]^ And the association between frailty and cancer mortality is inconsistent, with some studies showing an increased risk^[10,19,20]^ and others finding no associations.^[21]^ For PhenoAge, a study conducted in the United States involving 11,432 adults aged 20-84 years over 12.6 years found that for each 1 increment in PhenoAge, the corresponding HRs (95% CI) were 1.10 (1.07-1.13) for CVD mortality and 1.07 (1.05-1.09) for cancer mortality.^[6]^ In addition, the HR (95% CI) for CVD mortality per SD increase in PhenoAgeAccel was 1.46 (1.21-1.77), and the HR (95% CI) for cancer mortality was 1.21 (1.00-1.46) in ESTHER cohort study in Germany.^[18]^ In our study, we found that both FI and PhenoAgeAccel were associated with a higher risk of CVD mortality, whereas only PhenoAgeAccle was associated with increased cancer mortality risk. We did not observe an association between FI and cancer mortality, consistent with some previous studies.^[21,22]^ This observation may suggest that the predictive ability of FI for cancer mortality may be limited in the general population, while FI may be a useful predictor only in cancer patients.^[20,23,24]^

FI covers indicators of general health, diseases, and difficulties in activities of daily living, which makes it impossible to disentangle aging from disease.^[18]^ PhenoAge is not merely an indicator of disease or morbidity, but a marker for the cumulative impact of aging before clinical manifestations of diseases.^[6]^ Although C-reactive protein (CRP), a biomarker of frailty susceptibility,^[25,26]^ is included in the composition of PhenoAge, our study revealed a weak correlation between PhenoAge and FI (*r* = 0.24, *P* < 0.001). Although FI and PhenoAge both measure aspects of frailty and aging, they may capture different, potentially overlapping, pathophysiological processes. This point is particularly important as the associations we observed between FI, PhenoAge, and mortality risk may reflect complex interactions between these indices and various underlying biological mechanisms.

Despite lack of interaction between FI and PhenoAge, the joint use of FI and PhenoAge exhibited superior predictive capability compared to using either alone and individuals with a high FI and accelerated PhenoAge had the highest mortality risk. Such observations have important implications in identifying high-risk individuals for prevention and intervention. Further, the construction of FI and PhenoAge can be accomplished using existing data, without requiring additional, often cumbersome measurements. For instance, although there are many components in FI, most of the information can be easily extracted from routine clinical examinations and electronic health records.^[27,28]^ Similarly, PhenoAge can be obtained through regular clinical examinations and medical records. Among the available biological age evaluation metrics, clinical or phenotypic metrics, such as FI and PhenoAge, are considered to have superior performance, affordability, and utility.^[8,29,30]^

The strength of this study is the large sample size and national representativeness, comprehensive control of known and potential confounders, and robustness of the results in sensitivity analysis. Our study also has limitations. FI was derived from self-reported information, which may be subject to information bias. Participants might overestimate or underestimate their health conditions, particularly regarding chronic diseases and physical activity. The accuracy of PhenoAge could also be affected by measurement error or laboratory variability in the biomarkers used to derive the index. Moreover, while we controlled for known and potential confounders at baseline, residual confounding from unmeasured factors such as environmental influence cannot be ruled out. Changes in these confounding factors over time were not accounted for in the current analysis, and future studies should consider how these factors evolve during the study period. Both FI and PhenoAge were measured once at baseline, thus the trajectories and dynamic changes were not captured during follow-up.^[31, 32, 33]^ Finally, our study was not to develop a new predictive model, but rather to examine the associations of FI and PhenoAgeAccel with mortality outcomes, and to evaluate whether these indices, either individually or combined, provide added value in mortality risk prediction. It is important to note that while our study provides valuable insights into these associations, the results do not establish causal relationships. Future studies should explore these relationships further, with attention to the specific mechanisms that may link frailty and phenotypic aging to mortality risk.

In summary, our study suggests that both FI and PhenoAgeAccel were associated with a higher risk of mortality. Elevated FI and PhenoAgeAccel jointly forecasted a long-term increase in the risk of death. FI and PhenoAge have the potential to help identify high-risk individuals and guide targeted preventions and interventions. Future studies may need to examine the trajectories of FI and PhenoAge and their impacts on mortality risk.

## Supplementary Material

Supplementary Material Details
