# Supplementary material for: Joint association of the frailty index and phenotypic age with all-cause and cause-specific mortality: A prospective cohort study
Source: J Transl Int Med. 2026 Jun 13;14(3):436–45. doi: 10.1515/jtim-2026-0046 (PMC13320525; doi:10.1515/jtim-2026-0046)
Supplement: Supplementary file 1 — Supplementary Material Details [file jtim-2026-0046_sm.pdf]

Supplementary Materials

	Deaths	Deaths/1000 Py	HR (95%CI)
**CVD**			
Robust−Decelerated aging	56	1.59	1.00 (Ref)
Robust−Normal aging	91	4.23	1.11 (0.79−1.55)
Robust−Accelerated aging	62	18.81	2.16 (1.49−3.14)
Prefrail−Decelerated aging	136	1.39	1.17 (0.86−1.59)
Prefrail−Normal aging	277	8.35	1.52 (1.13−2.03)
Prefrail−Accelerated aging	197	16.86	1.99 (1.46−2.72)
Frail−Decelerated aging	39	2.06	1.56 (1.03−2.35)
Frail−Normal aging	143	12.11	2.14 (1.55−2.95)
Frail−Accelerated aging	194	28.23	2.66 (1.92−3.68)
**Cancer**			
Robust−Decelerated aging	64	1.82	1.00 (Ref)
Robust−Normal aging	67	4.57	0.88 (0.62−1.25)
Robust−Accelerated aging	18	5.46	0.69 (0.40−1.18)
Prefrail−Decelerated aging	139	1.42	0.88 (0.66−1.19)
Prefrail−Normal aging	221	6.66	1.16 (0.87−1.56)
Prefrail−Accelerated aging	110	9.41	1.13 (0.82−1.57)
Frail−Decelerated aging	43	2.27	1.32 (0.90−1.96)
Frail−Normal aging	99	8.38	1.39 (0.99−1.94)
Frail−Accelerated aging	98	14.26	1.31 (0.93−1.85)

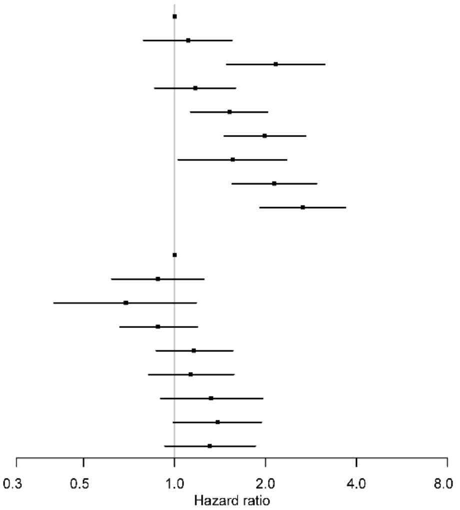

Supplementary Figure S1: The joint association of frailty status and PhenoAgeAccel with risk of death from CVD and cancer.

PYs: person-years; HR: hazard ratio; CI: confidence interval; Ref: reference; BMI: body mass index; CVD: cardiovascular disease. Multivariable models were adjusted for age, sex, ethnicity, marital status, education level, family income, smoking status, alcohol use, physical activity, healthy eating index score, BMI, and baseline prevalence of CVD and cancer.

Supplementary Table S1: Phenotypic age measures and Gompertz Coefficients.

Variable		Units	Weight
Albumin	Liver	g/L	-0.0336
Creatinine	Kidney	umol/L	0.0095
Glucose, serum	Metabolic	mmol/L	0.1953
C-reactive protein (log)	Inflammation	mg/dL	0.0954
Lymphocyte percent	Immune	%	-0.0120
Mean cell volume	Immune	fL	0.0268
Red cell distribution width	Immune	%	0.3306
Alkaline phosphatase	Liver	U/L	0.0019
White blood cell count	Immune	1000 cells/uL	0.0554
Age		Years	0.0804

Supplementary Table S2: List of 34 variables included in the frailty index.

	Deficits	Coding of variables
	Comorbidities*	
1	Angina/ angina pectoris	Yes = 1; No = 0
2	Arthritis	Yes = 1; No = 0
3	Broken hip	Yes = 1; No = 0
4	Cancer	Yes = 1; No = 0
5	Diabetes	Yes = 1; No = 0
6	High blood pressure	Yes = 1; No = 0
7	Heart attack	Yes = 1; No = 0
8	Heart disease	Yes = 1; No = 0
9	Osteoporosis	Yes = 1; No = 0
10	Stroke	Yes = 1; No = 0
11	Thyroid condition	Yes = 1; No = 0
12	Weak/failing kidneys	Yes = 1; No = 0
	Function	
13	Difficulty attending social event	Unable to do = 1; Some/much difficult = 0.5; Not difficult = 0
14	Difficulty dressing yourself	Unable to do = 1; Some/much difficult = 0.5; Not difficult = 0
15	Difficulty getting in and out of bed	Unable to do = 1; Some/much difficult = 0.5; Not difficult = 0
16	Difficulty grasping/holding small objects	Unable to do = 1; Some/much difficult = 0.5; Not difficult = 0
17	Difficulty lifting or carrying	Unable to do = 1; Some/much difficult = 0.5; Not difficult = 0
18	Difficulty managing money	Unable to do = 1; Some/much difficult = 0.5; Not difficult = 0
19	Difficulty preparing meals	Unable to do = 1; Some/much difficult = 0.5; Not difficult = 0
20	Difficulty standing up from armless chair	Unable to do = 1; Some/much difficult = 0.5; Not difficult = 0
21	Difficulty pushing or pulling large objects	Unable to do = 1; Some/much difficult = 0.5; Not difficult = 0
22	Difficulty stooping, crouching, kneeling	Unable to do = 1; Some/much difficult = 0.5; Not difficult = 0
23	Difficulty using fork and knife	Unable to do = 1; Some/much difficult = 0.5; Not difficult = 0
24	Difficulty walking between rooms on same floor	Unable to do = 1; Some/much difficult = 0.5; Not difficult = 0
	Signs/symptoms	
25	Confusion or inability to remember things	Yes = 1; No = 0
26	Cough regularly	Yes = 1; No = 0

	Deficits	Coding of variables
27	General hearing	Deaf = 1; Lot of trouble/Little trouble = 0.5; Good = 0
28	General vision	Yes = 1; No = 0
29	Leaked/ lost control of urine	Yes = 1; No = 0
	Others	
30	Frequency of healthcare use	⩾ 10 times = 1; 4-9 times = 0.5; 0-3 times = 0
31	Health compared to 1 year ago	Worse = 1; Better/Same = 0
32	Medications	⩾ 8 = 1; 4-7 = 0.5; 0-3 = 0
33	Overnight hospital stays	⩾ 3 times = 1; 1-2 times = 0.5; 0 times = 0
34	Self-reported health	Poor = 1; Fair = 0.5; Good = 0.25; Excellent/Very good = 0

*Comorbidities need to be diagnosed by doctors or certified by qualified hospitals.

Supplementary Table S3: Sensitivity analyses for frailty index and PhenoAgeAccel with all-cause and cause-specific mortality.

Items	Primary results HR (95% CI)	Sensitivity analyses results HR (95% CI)
**Number of participants**	20,089	19,643
**Frailty index**		
All-cause mortality		
Robust	1 (Ref)	1 (Ref)
Prefrail	1.53 (1.40-1.68)	1.49 (1.36-1.64)
Frail	2.86 (2.59-3.15)	2.72 (2.46-3.02)
Per 0.1 increment	1.41 (1.37-1.45)	1.39 (1.35-1.43)
Per 1 SD increment	1.47 (1.42-1.51)	1.44 (1.39-1.49)
CVD death		
Robust	1 (Ref)	1 (Ref)
Prefrail	1.31 (1.09-1.56)	1.28 (1.07-1.54)
Frail	1.90 (1.55-2.31)	1.97 (1.60-2.43)
Per 0.1 increment	1.25 (1.19-1.32)	1.28 (1.21-1.36)
Per 1 SD increment	1.28 (1.21-1.36)	1.31 (1.23-1.40)
Cancer death		
Robust	1 (Ref)	1 (Ref)
Prefrail	1.18 (0.99-1.42)	1.14 (0.94-1.39)
Frail	1.17 (0.94-1.45)	1.17 (0.93-1.47)
Per 0.1 increment	1.03 (0.97-1.10)	1.02 (0.96-1.10)
Per 1 SD increment	1.04 (0.97-1.11)	1.03 (0.95-1.11)
**PhenoAgeAccel**		
All-cause mortality		
Decelerated aging	1 (Ref)	1 (Ref)
Normal aging	1.42 (1.29-1.55)	1.36 (1.24-1.50)
Accelerated aging	2.94 (2.66-3.26)	2.85 (2.56-3.17)
Per 1 increment	1.05 (1.04-1.05)	1.05 (1.04-1.05)
Per 1 SD increment	1.41 (1.38-1.45)	1.41 (1.37-1.45)
CVD death		
Decelerated aging	1 (Ref)	1 (Ref)
Normal aging	1.22 (1.04-1.43)	1.24 (1.04-1.46)
Accelerated aging	1.75 (1.46-2.10)	1.81 (1.49-2.20)
Per 1 increment	1.03 (1.02-1.03)	1.03 (1.02-1.03)
Per 1 SD increment	1.21 (1.15-1.27)	1.23 (1.17-1.30)
Cancer death		
Decelerated aging	1 (Ref)	1 (Ref)
Normal aging	1.18 (0.98-1.43)	1.11 (0.91-1.36)
Accelerated aging	1.49 (1.20-1.86)	1.40 (1.11-1.76)
Per 1 increment	1.02 (1.01-1.02)	1.01 (1.00-1.02)
Per 1 SD increment	1.13 (1.07-1.20)	1.10 (1.04-1.17)

Multivariable models were adjusted for age, sex, ethnicity, marital status, education level,

family income, smoking status, alcohol use, physical activity, healthy eating index score, BMI, and baseline prevalence of CVD and cancer (only in corresponding cause-specific analyses). HR: hazard ratio; CI: confidence interval; Ref: reference; SD: standard deviation; CVD: cardiovascular disease; BMI: body mass index.

Supplementary Table S4: Additive and multiplicative interactions between frailty index and PhenoAgeAccel on all-cause and cause-specific deaths.

Items	Additive interactions		Multiplicative interactions
	**RERIs (95% CIs)**	**APs (95% CIs)**	***P* for multiplicative interactions**
All-cause mortality	0.148 (0.078-0.219)	0.265 (0.109-0.422)	0.054
CVD	0.168 (-0.017-0.353)	0.285 (-0.120-0.689)	0.743
Cancer	-0.004 (-0.181-0.172)	-0.007 (-0.292-0.278)	0.157

Additive interactions were estimated using RERIs and APs with their 95% CIs (estimated by variance recovery method) by fitting cox hazard models with an interaction term for frailty index and PhenoAgeAccel on all-cause and cause-specific deaths. Multiplicative interactions were tested by comparing the multivariate-adjusted models with and without cross-product interaction terms using likelihood-ratio tests. RERI: relative excess risk due to interaction; AP: attributable proportion due to interaction; CI: confidence interval; CVD: cardiovascular disease.

Supplementary Table S5: 10-year cumulative mortality risk and absolute risk differences across joint groups.

Joint groups	10-years cumulative mortality risk, %	10-year absolute risk differences, % (95% CI)	*P*
Robust-Decelerated aging	5.53	-	-
Robust-Normal aging	16.77	11.24 (8.43-14.04)	< 0.001
Robust-Accelerated aging	30.7	25.16 (19.22-31.1)	< 0.001
Prefrail-Decelerated aging	4.93	-0.61 (-1.84-0.63)	0.67
Prefrail-Normal aging	20.74	15.2 (13.12-17.29)	< 0.001
Prefrail-Accelerated aging	38.8	33.26 (29.84-36.69)	< 0.001
Frail-Decelerated aging	7.99	2.46 (0.59-4.33)	< 0.001
Frail-Normal aging	31.87	26.34 (22.89-29.79)	< 0.001
Frail-Accelerated aging	58.79	53.26 (49.05-57.47)	< 0.001

Supplementary Table S6: Sensitivity analyses for joint association of frailty index and PhenoAgeAccel with all-cause and cause-specific mortality.

Items	Primary results HR (95% CI)	Sensitivity analyses results HR (95% CI)
**Number of participants**	20,089	19,643
**All causes mortality**		
Robust-Decelerated aging	1 (Ref)	1 (Ref)
Robust-Normal aging	1.25 (1.04-1.49)	1.21 (1.01-1.45)
Robust-Accelerated aging	2.08 (1.68-2.59)	1.96 (1.56-2.46)
Prefrail-Decelerated aging	1.17 (0.99-1.39)	1.12 (0.94-1.33)
Prefrail-Normal aging	1.75 (1.50-2.05)	1.63 (1.39-1.92)
Prefrail-Accelerated aging	3.01 (2.55-3.55)	2.82 (2.37-3.34)
Frail-Decelerated aging	2.19 (1.77-2.70)	2.15 (1.73-2.67)
Frail-Normal aging	3.35 (2.82-3.97)	3.21 (2.69-3.83)
Frail-Accelerated aging	5.32 (4.50-6.29)	4.90 (4.12-5.84)
**CVD**		
Robust-Decelerated aging	1 (Ref)	1 (Ref)
Robust-Normal aging	1.11 (0.79-1.55)	1.03 (0.73-1.44)
Robust-Accelerated aging	2.16 (1.49-3.14)	2.00 (1.36-2.96)
Prefrail-Decelerated aging	1.17 (0.86-1.59)	1.09 (0.80-1.50)
Prefrail-Normal aging	1.52 (1.13-2.03)	1.40 (1.04-1.89)
Prefrail-Accelerated aging	1.99 (1.46-2.72)	2.06 (1.50-2.85)
Frail-Decelerated aging	1.56 (1.03-2.35)	1.51 (0.99-2.32)
Frail-Normal aging	2.14 (1.55-2.95)	2.23 (1.53-2.96)
Frail-Accelerated aging	2.66 (1.92-3.68)	2.59 (1.85-3.63)
**Cancer**		
Robust-Decelerated aging	1 (Ref)	1 (Ref)
Robust-Normal aging	0.88 (0.62-1.25)	0.92 (0.65-1.32)
Robust-Accelerated aging	0.69 (0.40-1.18)	0.68 (0.39-1.22)
Prefrail-Decelerated aging	0.88 (0.66-1.19)	0.87 (0.63-1.18)
Prefrail-Normal aging	1.16 (0.87-1.56)	1.09 (0.81-1.48)
Prefrail-Accelerated aging	1.13 (0.82-1.57)	1.13 (0.80-1.60)
Frail-Decelerated aging	1.32 (0.90-1.96)	1.30 (0.86-1.95)
Frail-Normal aging	1.39 (0.99-1.94)	1.29 (0.90-1.84)
Frail-Accelerated aging	1.31 (0.93-1.85)	1.27 (0.88-1.84)

Multivariable models were adjusted for age, sex, ethnicity, marital status, education level, family income, smoking status, alcohol use, physical activity, healthy eating index score, BMI, and baseline prevalence of CVD and cancer (only in corresponding cause-specific analyses). HR: hazard ratio; CI: confidence interval; Ref: reference; CVD: cardiovascular disease; BMI: body mass index.

Supplementary Table S7: Comparison of the value of frailty index and PhenoAgeAccel over and above their respective values in risk prediction.

Model	C-index (95% CI)	*P*	NRI, continuous (95% CI), %	*P*	IDI (95% CI), %	*P*
Conventional model* + FI (Reference)	0.875 (0.870-0.880)	-	-	-	-	-
Conventional model + PhenoAgeAccel	0.877 (0.872-0.882)	0.007	-0.21 (-10.94-11.35)	0.55	0.30 (-0.30-0.90)	0.28
Conventional model + FI + PhenoAgeAccel	0.882 (0.877-0.887)	< 0.001	26.29 (19.63-35.68)	< 0.001	1.70 (1.30-2.10)	< 0.001

Conventional model included age, sex, ethnicity, marital status, education level, family income, smoking status, alcohol, physical activity, healthy eating index score, and BMI. CI: confidence interval; NRI=: net reclassification improvement; IDI: integrated discrimination index; FI: frailty index; BMI: body mass index.
